# Long-term outcomes in high-risk patients with non-ST-segment elevation myocardial infarction

**DOI:** 10.1007/s11239-015-1227-1

**Published:** 2015-05-23

**Authors:** Marc Cohen

**Affiliations:** Division of Cardiology, Newark Beth Israel Medical Center, 201 Lyons Avenue, Newark, NJ 07112 USA

**Keywords:** Acute coronary syndromes, Dual antiplatelet therapy, Non-ST-segment elevation myocardial infarction

## Abstract

Greater use of evidence-based therapies has improved outcomes for patients with acute coronary syndromes (ACS) in recent decades. Consequently, more ACS patients are surviving beyond 12 months; however, limited data exist to guide treatment in these patients. Long-term outcomes have not improved in non-ST-segment elevation myocardial infarction (NSTEMI) patients at the same rate seen in ST-segment elevation myocardial infarction patients, possibly reflecting NSTEMI patients’ more complex clinical phenotype, including older age, greater burden of comorbidities and higher likelihood of a previous myocardial infarction (MI). This complexity impacts clinical decision-making, particularly in high-risk NSTEMI patients, in whom risk–benefit assessments are problematical. This review examines the need for more effective long-term management of NSTEMI patients who survive ≥12 months after MI. Ongoing risk assessment using objective measures of risk (for bleeding and ischemia) should be used in all post-MI patients. While 12 months appears to be the optimal duration of dual antiplatelet therapy for most patients, this may not be the case for high-risk patients, and more research is urgently needed in this population. A recent subgroup analysis from the DAPT study in patients with or without MI who had undergone coronary stenting (31 % presented with MI; 53 % had NSTEMI) and the prospective PEGASUS-TIMI 54 trial in patients with a prior MI and at least one other risk factor (40 % had NSTEMI) demonstrated that long-term dual antiplatelet therapy improved cardiovascular outcomes but increased bleeding. Further studies will help clarify the role of dual antiplatelet therapy in stable post-NSTEMI patients.

## Introduction

Approximately 50–75 % of patients experiencing an acute coronary event in the US each year have a non-ST-segment elevation myocardial infarction (NSTEMI) [1–3], and the proportion of NSTEMI events is increasing [4]. Mortality rates after myocardial infarction (MI) have decreased over the last 20 years [3], but improvement in outcomes differs between ST-segment elevation myocardial infarction (STEMI) and NSTEMI patients. Compared with STEMI patients, NSTEMI patients have lower short-term mortality rates, and higher rates of long-term mortality, even after adjustment for risk factors [2, 4]. One-year mortality rates for STEMI patients have declined recently, but in NSTEMI patients, the trend is inconsistent and less marked [3]. Registry data suggest that the 10-year survival rate after NSTEMI is around 50 % [5].

Several years ago, the only interventional options for acute coronary syndromes (ACS) were balloon angioplasty or surgery, and post-percutaneous coronary intervention (PCI) patients received warfarin. PCI provided only moderate benefit [6], but the advent of coronary stents and more potent antiplatelet agents have significantly improved outcomes, such that PCI is now recommended for most NSTEMI patients [7, 8]. Evidence-based therapy use, during and after hospitalization, has increased over the past several years in both STEMI and NSTEMI patients [3]. Yet, despite this increase and the associated improvement in outcomes after ACS, a significant residual risk, in the short (up to 12 months) and long term (≥3 years or longer), for cardiovascular-related death remains [9].

A key question is why are long-term outcomes not improving in NSTEMI patients as for STEMI patients? The likely reason is that patients with NSTEMI tend to have a more complex clinical phenotype. Compared with STEMI patients, NSTEMI patients tend to be older, have more comorbidities, are more likely to have an MI history, and to experience recurrent ischemia after the acute event [2, 3]. Thus, clinical decision making is more complicated, particularly in high-risk NSTEMI patients, as risk–benefit assessment is less straightforward. Poorer outcomes in high-risk NSTEMI populations indicate the need for more effective treatments. Clear evidence supports treatment decisions in the acute and post-acute phase of ACS; however, data are limited to guide long-term management in the growing population of patients who survive beyond 12 months after an event– many of whom are elderly and have comorbidities.

This review examines the need for more effective management; reviews the evidence and rationale for treatment; identifies opportunities to improve outcomes; and outlines recent research addressing unmet needs in high-risk NSTEMI patients. Herein, ‘long term’ refers to outcomes occurring ≥12 months after the initial ACS.

## Current treatment recommendations for high-risk patients

An urgent invasive strategy is generally preferred as initial management in NSTEMI patients with refractory angina, signs or symptoms of heart failure, and hemodynamic instability [7]. Patients should undergo angiography within 2 h of admission, with appropriate anti-ischemic, antiplatelet, and anticoagulant therapy [7]. Urgent catheterization is preferred in high-risk patients, who show better outcomes with this approach versus delayed catheterization [10]. Other high-risk NSTEMI patients should undergo an early invasive strategy (within 24 h of presentation) [7]. For in-hospital NSTEMI management, currently recommended dual antiplatelet therapy incorporates aspirin with either clopidogrel or ticagrelor; ticagrelor is preferred in patients undergoing early invasive or ischemia-guided therapy [7].

Previously, the American Heart Association/American College of Cardiology (AHA/ACC) guidelines suggested that any P2Y_12_ inhibitor could be considered in NSTEMI patients, but the 2014 update [7] brings antiplatelet recommendations more in line with European guidelines. European guidelines recommend ticagrelor for all patients at moderate to high risk of ischemic events, regardless of initial treatment strategy [8]. In Europe, prasugrel is recommended for P2Y_12_ inhibitor-naïve patients with known coronary anatomy who are proceeding to PCI, unless contraindicated, or patients at high risk of bleeding [8]. Clopidogrel is recommended only for patients who cannot receive ticagrelor or prasugrel [8].

The 2014 AHA/ACC NSTEMI guidelines emphasize secondary prevention, including ongoing use of dual antiplatelet therapy for post-hospital care [7]. Table 1 summarizes the AHA/ACC recommendations for maintenance dosing of antiplatelet agents. As with US guidelines, European guidelines recommend treatment with a P2Y_12_ inhibitor for at least 12 months after the event [8].

**Table 1:** 2014 AHA/ACC guidelines for use of antiplatelet agents in patients with invasively or non-invasively managed NSTEMI [7]

AHA/ACC recommendations	COR	LOE
Duration and maintenance dose of P2Y_12_ receptor inhibitor therapy in NSTEMI patients undergoing an early invasive or ischemia-guided strategy		
Clopidogrel 75 mg daily or ticagrelor^a^ 90 mg twice daily should be given for up to 12 months	I	B
It is reasonable to choose ticagrelor over clopidogrel	IIa	B
Duration and maintenance dose of P2Y_12_ receptor inhibitor therapy in NSTEMI patients who underwent PCI and received a stent		
Clopidogrel 75 mg daily, prasugrel^b^ 10 mg daily, or ticagrelor 90 mg twice daily should be given for at least 12 months	I	B
It is reasonable to choose prasugrel over clopidogrel in patients who are not at high risk of bleeding complications	IIa	B
If the risk of morbidity from bleeding outweighs the anticipated benefits after stent implantation, earlier discontinuation of P2Y_12_ receptor is reasonable	IIa	C
Continuation of dual antiplatelet therapy beyond 12 months may be considered in patients undergoing stent implantation	IIb	C

*COR* class of recommendation, *LOE* level of evidence, *NSTEMI* non-ST-elevation myocardial infarction, *PCI* percutaneous coronary intervention

^a^The recommended maintenance dose of aspirin to be used with ticagrelor is 81 mg daily

^b^Prasugrel should not be administered to patients with a prior history of stroke or transient ischemic attack (COR: III; LOE: B)

All post-NSTEMI patients should receive beta-blockers and statins long term, unless contraindicated [7]; patients may also require medications to modify risk factors, such as antihypertensive medications to achieve target blood pressure, angiotensin-converting enzyme (ACE) inhibitors for left ventricular dysfunction, and antihyperglycemic agents to maintain HbA1c <7 % [7, 11].

## Data supporting current recommendations

**Fig. 1:**
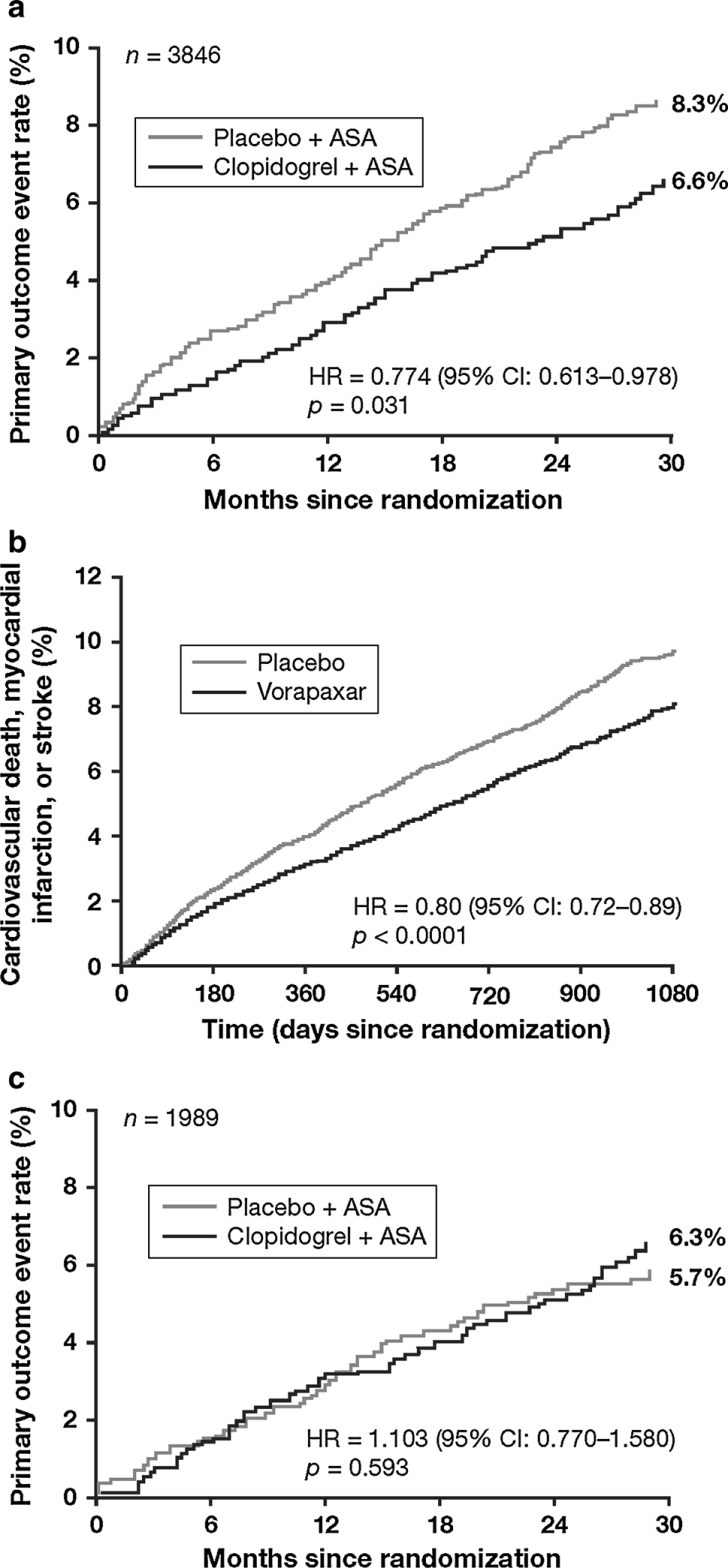
Kaplan–Meier curves for the primary composite end point of cardiovascular death, MI, or stroke in subgroups of patients. **a** Patients with prior MI in CHARISMA [13]; **b** Patients with prior MI in the TRA2°P-TIMI 50 trial [9]; **c** Patients with established coronary artery disease who had not had an MI in CHARISMA [13]. *ASA* aspirin, *CI* confidence interval, *HR* hazard ratio Panels a + c are reprinted from J Am Coll Cardiol 49 (19), Bhatt DL et al. ‘Patients with prior myocardial infarction, stroke, or symptomatic peripheral arterial disease in the CHARISMA trial.’ 1982–1988, copyright (2007), with permission from Elsevier. Panel b is reprinted from The Lancet 380, Scirica BM et al. ‘Vorapaxar for secondary prevention of thrombotic events for patients with previous myocardial infarction: a prespecified subgroup analysis of the TRA2°P-TIMI 50 trial.’ 1317–1324, copyright (2012) with permission from Elsevier

CHARISMA assessed clopidogrel plus aspirin in a high-risk cohort of patients with established atherothrombotic disease or at high risk of atherothrombosis [12]. Although the group with risk factors did not necessarily derive clinical benefit from dual antiplatelet therapy, those with established disease did [12]. Therefore, a subanalysis of the high-risk secondary prevention population was undertaken [13]. These patients had prior MI (*n* = 3846), stroke (*n* = 3245), or symptomatic peripheral arterial disease (PAD) (*n* = 2838), and received clopidogrel or placebo, plus aspirin, for a median of 27.6 months. Patients taking clopidogrel plus aspirin had a significantly lower risk of cardiovascular death, MI, or stroke (primary end point) versus those receiving placebo plus aspirin (7.3 vs. 8.8 %; hazard ratio [HR], 0.83 [95 % confidence interval (CI), 0.72–0.96]; *p* = 0.01). In prior MI cohort, the primary composite end point occurred in 6.6 % of patients taking clopidogrel plus aspirin versus 8.3 % of those taking placebo plus aspirin (HR, 0.774 [95 % CI, 0.613–0.978]; *p* = 0.031). The benefit of dual antiplatelet therapy was not seen in patients with established coronary artery disease (CAD) without prior MI (Fig. 1) [13], implying that dual antiplatelet therapy may provide a benefit in the post-MI setting, even if initiated some time after the event.

Current recommendations do not provide clear clinical guidance on the duration of dual antiplatelet therapy, or whether the therapy duration depends on the patient’s risk profile. Previous studies (including two randomized controlled trials) showed no significant benefit in continuing dual antiplatelet therapy beyond 12 months in patients who have undergone PCI and survive event-free for 1 year [14–16]. A meta-analysis of the randomized data showed the overall odds ratio for interrupting dual antiplatelet therapy at 12 months versus continuing therapy was 1.18 (95 % CI, 0.61–2.29 [*p* = 0.62]) [15]. However, these studies were underpowered, and did not necessarily enroll high-risk patients [14, 15], so the question of whether or not extended treatment would be beneficial in high-risk patients remains unanswered.

## Impact of risk factors on outcomes

Optimal, evidence-based treatment after NSTEMI can only reduce the risk of an event. Even optimally treated patients face a residual risk of adverse cardiovascular outcomes due to the underlying disease process, their general health, and any comorbid conditions. So what constitutes ‘high risk’ after NSTEMI?

In the last decade, much has been learned about long-term risk from registry studies, including GRACE, ACTION, GTWG database, and CRUSADE registry data of NSTEMI patients. These registries have contributed to the development of a number of risk assessment tools (see below). A consistent finding is that risk of adverse outcomes increases with older age, male gender, diabetes, worse renal function, renal failure, anemia, prior vascular disease/CAD, heart failure (past or present), poor hemodynamics at presentation, and clinical instability during ACS [17–20].

The Worcester Heart Attack Study (WHAS) investigated long-term risk factors for mortality after discharge specifically in NSTEMI patients, and found that older age, male gender, longer hospital stay, history of stroke, heart failure, or diabetes, and stroke or heart failure during hospitalization all predicted long-term mortality [2]. Patients were studied for up to 5 years after the index event, but analyses did not distinguish between risk factors for death ≤12 versus >12 months [2].

Risk factors for 10-year mortality after NSTEMI in the PRAIS-UK registry were age, ST depression or bundle branch block on initial electrocardiogram (ECG), and a history of heart failure [5]. However, this analysis was based on UK NSTEMI patients in 1998 and 1999, and may not be applicable to a contemporary NSTEMI population either in the UK or elsewhere. EPICOR developed a risk score for mortality in patients with STEMI (*n* = 4943) and non-ST-elevation ACS (*n* = 5625) [21]. Twelve independent predictors of mortality were identified. In order of importance, these were: age, lower ejection fraction, poorer EQ-5D quality of life, elevated serum creatinine, in-hospital cardiac complications, chronic obstructive pulmonary disease, elevated blood glucose, male gender, no PCI/coronary artery bypass grafting (CABG) after NSTE-ACS, low hemoglobin, PAD, and on diuretics at discharge. However, the risk score was based on 12-month mortality risk and may not be applicable to long-term outcomes.

Evidence suggests that biomarkers may be useful to help identify high-risk patients after NSTEMI. Recent research identified a number of biomarkers that enhance the accuracy of the GRACE risk assessment in NSTEMI patients, including B-type natriuretic peptide [22], C-terminal vasopressin or copeptin [23], and growth differentiation factor-15 [24]. However, none of these biomarkers has yet been adopted for risk assessment during clinical practice.

In summary, patients at high risk after NSTEMI are likely to be of older age, men, and have cardiovascular (e.g. heart failure, stroke) and non-cardiovascular (e.g. poor renal function, diabetes) comorbidities, and a poorer quality of life than those at low risk.

## Determination of risk

Several studies indicate that when established risk assessment methods are not used, physicians tend to underestimate risk in high-risk patients and overestimate risk in low-risk patients [25]. Additionally, physicians tend to estimate risk based on the intensity of treatment received during the ACS [25, 26]. In particular, physicians underestimate risk associated with age, and may view younger ACS patients as having a more aggressive disease phenotype than older patients, while underestimating the impact of age-associated accumulated coronary artery damage [26]. Therefore, it is important that physicians use validated objective measures of risk when assessing ACS patients.

### Ischemic risk

Several risk-scoring tools evaluated the risk of subsequent events in ACS patients, some of which can be used in NSTEMI patients (Table 2) [17–19, 27–30]. These risk scores, derived mainly from randomized controlled trials and registry data, assess a patient’s short- to medium-term risk of an adverse outcome (usually death and/or nonfatal MI).

**Table 2:** Risk stratification tools for use in patients with NSTEMI

	PREDICT [17]	TIMI [27]	PURSUIT [28]	FRISC II [29]	GRACE [18]	SYNERGY [30]	CRUSADE [19]
Relevant population	MI or UA	UA or NSTEMI	UA or NSTEMI	UA or NSTEMI	Any ACS	NSTEMI or UA	NSTEMI aged ≥65 years
Outcome	Death	Death, MI or urgent revascularization	Death or nonfatal MI	Death or death/MI	Death or death/MI	Death	Mortality
Time horizon	30 days, 2 years, 6 years	14 days	30 days	1 year	6 months	From 30 days to 1 year	1 year
Data source	Observational cohort study	RCT	RCT	RCT	Registry	RCT	Registry
Items included	Age	Age	Age	Age	Age	Age	Age
	History of CVD^b^	Having ≥3 CHD risk factors^a^	Gender	Gender	HR	Creatinine clearance	Serum creatinine
	CHF at presentation Shock at presentation Kidney function ECG severity score (based on presence of Q wave, ST-segment deviation, bundle branch block) Charlson comorbidity index score	Known CHD (≥50 % stenosis) Aspirin use in past 7 days Severe angina (≥2 episodes in 24 h) ECG changes of ≥0.5 mm Positive biomarker	Worst CCS class in previous 6 weeks HR SBP Signs of heart failure ST depression on presenting ECG	Diabetes Previous MI ST depression on admission Elevated biomarkers	SBP Serum creatinine Killip class Cardiac arrest at admission ST-segment deviation Elevated cardiac enzymes/markers	Weight Baseline hemoglobin level Baseline platelet count Nadir platelet count Atrial fibrillation/flutter Statin use in first 30 days since initial hospitalization CABG during first 30 days after initial hospitalization Gender	SBP Signs of heart failure on presentation HR Weight Prior heart failure Hematocrit Troponin ratio Prior stroke Diabetes Gender Prior PAD

TIMI risk score for UA/NSTEMI: http://www.mdcalc.com/timi-risk-score-for-uanstemi/

GRACE risk score for ACS: http://www.mdcalc.com/grace-acs-risk-and-mortality-calculator/

*ACS* acute coronary syndrome, *CABG* coronary artery bypass grafting, *CCS* Canadian Cardiovascular Society (angina measure), *CHD* coronary heart disease, *CHF* congestive heart failure, *CVD* cardiovascular disease, *DM* diabetes mellitus, *ECG* electrocardiogram, *HR* heart rate, *MI* myocardial infarction, *NSTEMI* non-ST-segment elevation myocardial infarction, *PAD* peripheral arterial disease, *RCT* randomized controlled trial, *SBP* systolic blood pressure, *UA* unstable angina

^a^Family history of CHD, hypertension, hypercholesterolemia, diabetes, or current smoker

^b^History of MI, stroke, angina >8 weeks before admission, CABG, cardiac arrest and/or hypertension

While no risk tool is clearly superior to another, an analysis by the UK National Institute for Health and Clinical Excellence (NICE) suggests that the PURSUIT, GRACE, and PREDICT tools provide better discrimination of mortality risk than the Thrombolysis in Myocardial Infarction (TIMI) score [31]. TIMI and GRACE risk scores are most commonly used, and available as online calculators to simplify risk stratification in clinical practice [32].

Few of these tools were designed to assess long-term risk (past 1 year). However, the GRACE score was a useful predictor of death at 5 years in a mixed ACS population in UK and Belgian GRACE registries [33], and at 10 years in an NSTEMI cohort from the PRAIS UK registry [5].

The PREDICT tool was one of the few designed to predict long-term outcomes, and has a better predictive power for 2- or 6-year outcomes than for 30-day outcomes [17]. However, PREDICT was developed in a mostly white population, and is not specific for NSTEMI patients.

The SYNERGY tool was designed to assess 1-year outcomes in patients surviving 30 days after the acute event [30]. This tool may be particularly useful for the care of long-term, post-hospitalization NSTEMI patients because it excludes risk factors that predict death during the immediate post-ACS period.

### Bleeding risk

Few tools exist to assess bleeding risk; the most widely used was developed from the CRUSADE registry data of NSTEMI patients, and is designed to assess risk of in-hospital bleeding. The CRUSADE bleeding risk assessment tool assigns a score based on the patient’s baseline hematocrit, creatinine clearance, heart rate, gender, systolic blood pressure, and presence of prior vascular disease, congestive heart failure on presentation or diabetes mellitus [34].

Another bleeding risk assessment tool was developed from the ACTION-GTWG database [35], including STEMI and NSTEMI patients. The ACTION-GTWG bleeding-risk score is more complicated than CRUSADE, and includes 12 variables. It includes all of the variables in the CRUSADE risk tool (notwithstanding using hemoglobin instead of hematocrit as a measure of anemia, and serum creatinine instead of creatinine clearance for renal function), and also includes body weight, warfarin use, and the presence and type of ST changes on ECG [35]. This tool has been validated for the prediction of major bleeding during hospitalization, but no data are available on its use to predict long-term bleeding.

### Balancing the risk of ischemic versus bleeding events

Treatment selection in clinical practice must balance risk of ischemic events with bleeding risk, which is difficult in patients with multiple risk factors. Many risk factors for ischemic events are the same as for bleeding events, complicating decision making. Bleeding during hospitalization for NSTEMI is associated with a higher rate of mortality in the first 30 days, 1 and 3 years after an event, particularly in patients undergoing PCI [36]. This finding may be partly explained by reduced use of dual antiplatelet therapy at discharge in patients with a bleeding event during hospitalization [36].

While bleeding may be a marker for a poor long-term outcome, bleeding may not be causally related to outcome [37]. In an analysis of CHARISMA (overall cohort) comparing patients who continued dual antiplatelet therapy and those who discontinued, the rate of adverse outcomes, both cardiovascular and bleeding events, over 28 months was higher in those who discontinued versus continued antiplatelet therapy [38]. The increased bleeding rate among patients discontinuing dual antiplatelet therapy was ascribed to patients who discontinued being more likely to have ischemic risk factors, such as age and history of significant cardiovascular disease; additionally, therapy may have been discontinued because of prior bleeding [38].

For many high-risk patients, the risk-benefit profile is not clear-cut, and should be considered on an individual basis. For example, triple therapy (dual antiplatelet therapy plus an oral anticoagulant) has been evaluated in ACS patients. The ATLAS-ACS 2 TIMI-51 trial evaluated rivaroxaban (a selective factor Xa inhibitor) versus placebo in ACS patients; all patients received aspirin plus a thienopyridine [39]. Rivaroxaban reduced the rate of the composite end point of MI, stroke, or death from cardiovascular causes versus placebo (8.9 vs. 10.7 %; HR, 0.84 [95 % CI, 0.74–0.96]; *p* = 0.008). However, versus placebo, rivaroxaban also increased rates of major bleeding not related to CABG (2.1 vs. 0.6 %, *p* < 0.001) and intracranial hemorrhage (0.6 vs. 0.2 %, *p* = 0.009), without a significant increase in fatal bleeding (0.3 vs. 0.2 %, *p* = 0.66) [39]. These data suggest that, in some patients, the increased risk of major bleeding associated with triple therapy may outweigh the benefit associated with a reduction in ischemic events [39], reinforcing the importance of careful risk assessment in each patient based on clinical and demographic characteristics.

## Are patients being treated on the basis of risk?

Early data from the CRUSADE registry showed that dual antiplatelet therapy was underutilized at discharge in NSTEMI patients, and underutilization was greater in some patient subgroups—those not undergoing PCI, those aged >75 years, women, and Hispanic patients [40]. Fewer than 50 % of eligible NSTEMI patients not undergoing PCI received dual antiplatelet therapy in the CRUSADE registry in 2002/2003. This observation was also true in the ACTION registry (2007–2010), which showed that 40.7 % of NSTEMI patients in the US not undergoing PCI received dual antiplatelet therapy [41]. This proportion is lower than in NSTEMI patients not undergoing PCI reported in the UK or Swedish registries during the same period (70.6 and 48.8 %, respectively) [41]. However, the overall rate of discharge antiplatelet use among NSTEMI patients reported in the ACTION registry was around 74 % between 2009 and 2012 [42].

**Fig. 2:**
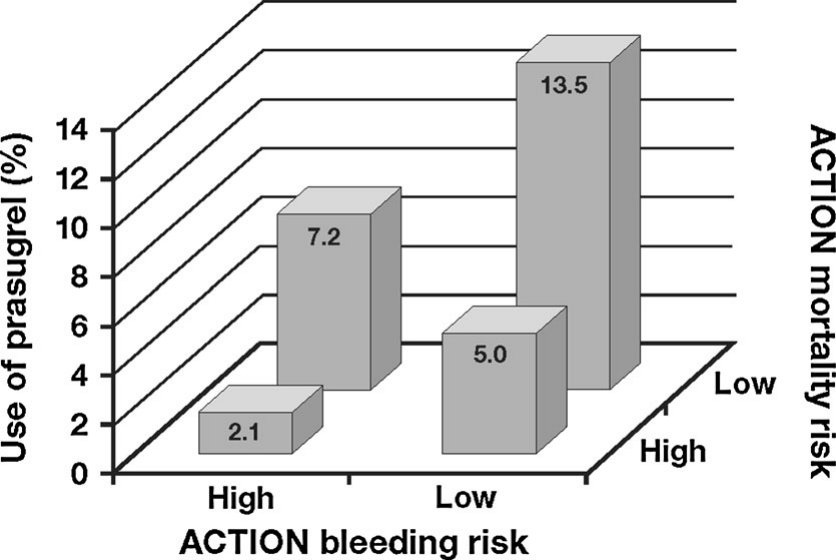
Prasugrel use by mortality and bleeding risk in the NSTEMI population of the ACTION registry [42]. Reproduced from Sherwood MW et al. ‘Early clopidogrel versus prasugrel use among contemporary STEMI and NSTEMI patients in the US: insights from the National Cardiovascular Data Registry. J Am Heart Assoc 2014;3:e000849, with permission from Wiley. ©2014 The Authors. Published on behalf of the American Heart Association, Inc., by Wiley Blackwell

An interesting finding in the recent analysis of ACTION registry data (2009–2012) is the use of prasugrel in patients in whom it is not indicated, or should be used with caution. Prasugrel was used in 2 % of medically managed patients, 2 % of patients aged ≥75 years, and 5 % of patients weighing <60 kg [42]. In addition, the highest rate of prasugrel use was in patients with the lowest risk of ischemic or bleeding events (Fig. 2) [42], despite the fact that evidence supports its use in individuals at high risk of ischemic events.

Collectively, these data suggest that high-risk patients may be undertreated after NSTEMI. Just as clinicians tend to underestimate the ischemic risk in high-risk patients [25], it is possible that they may be overestimating the risk of bleeding, as has been shown with the use of antithrombotic therapy in high-risk atrial fibrillation patients [43]. Minimizing the risk of bleeding is important, but undertreating may increase the risk of ischemic events. Ischemia has irreversible effects on tissue and may have long-term sequelae (e.g. heart failure, stroke-related disability), whereas bleeding can almost always be controlled and, with the exception of intracerebral bleeding, does not generally have long-lasting effects.

## Treatment options: current knowledge and future research

As more patients survive ACS, more evidence is needed to support long-term treatment decisions, but few studies have investigated outcomes and strategies for ≥12 months after an event. The TRA2°P-TIMI 50 trial investigated the effect of the protease-activated receptor (PAR)-1 antagonist voraxapar versus placebo on cardiovascular outcomes (composite end point: cardiovascular death, MI, or stroke) in patients with a history of atherothrombosis [44]. The patient cohort included a large subgroup with a history of MI (*n* = 17 779), but these patients had the qualifying MI between 2 weeks and 12 months prior to enrolment [9]. In these patients with a history of MI (all of whom were taking aspirin), voraxapar significantly reduced the 3-year risk of the composite end point by 20 %, compared with placebo (HR, 0.80 [95 % CI, 0.72–0.89]; *p* < 0.0001), but at the expense of a significant increase in the risk of moderate or severe bleeding (HR, 1.61 [95 % CI, 1.31–1.97]; *p* < 0.0001) [9]. When reviewed alongside the data from CHARISMA, these data suggest that dual antiplatelet therapy may be an effective strategy in stable post-MI patients, and provides continued risk reduction when continued for longer than 12 months (Fig. 1). However, the data are not specific to NSTEMI patients and provide little guidance on the effect of treatment started ≥12 months after MI.

The APOLLO study uses electronic medical record data from patients who survive the first 12 months after an ACS to evaluate their subsequent outcomes. This large-scale study is being conducted in Sweden, England, France, and the US, and will provide important information about outcomes in a ‘real-world,’ unselected patient cohort representative of clinical practice. Preliminary data suggested that in the US, outcome rates over 3 years were worse than those in the studied European countries. For example, the all-cause mortality rate over 3 years was 30.2 % in the US, compared with 20.1 % in Sweden, 14.3 % in France, and 13.7 % in the UK [45]. However, US patients also had more comorbidities than patients in other countries, and the difference was less marked (although significant vs. Sweden and UK) after adjustment for risk factors; adjusted all-cause mortality was 12.8 % in the US, compared with 12.4 % in France, 11.2 % in Sweden, and 8.7 % in the UK [45]. The DAPT study investigated the incidence of stent thrombosis and major cardiovascular and cerebrovascular events (a composite of death, MI, or stroke) in patients who had received a stent [46]. In this randomized controlled trial, 9961 patients received thienopyridine therapy (clopidogrel or prasugrel) for 12 months, and were then randomly assigned to either continue thienopyridine therapy or receive placebo for 18 months. Rates of stent thrombosis in the thienopyridine group were reduced versus placebo (0.4 vs. 1.4 %; HR, 0.29 [95 % CI, 0.17–0.48]; *p* < 0.001), as were composite end-point events (4.3 vs. 5.9 %; HR, 0.71 [95 % CI, 0.59–0.85]; *p* < 0.001) and MI (2.1 vs. 4.1 %; HR, 0.47; *p* < 0.001). However, all-cause mortality was higher in the group that continued thienopyridine treatment, compared with placebo (2 vs. 1.5 %; HR, 1.36 [95 % CI, 1.00–1.85]; *p* = 0.05). The primary safety end point (rate of moderate or severe bleeding) was higher in the thienopyridine group versus placebo (2.5 vs. 1.6 %, *p* = 0.001). A recent subgroup analysis from the DAPT study examined these same efficacy and safety end points among patients undergoing coronary stenting after presentation with or without an acute MI (*n* = 3576 presented with MI [31 %]; 53 % had NSTEMI) [47]. Compared with placebo, long-term thienopyridine therapy statistically significantly reduced the occurrence of stent thrombosis in both patient subgroups and significantly reduced major adverse cardiovascular and cerebrovascular events to a greater degree in the MI group, but with a significantly higher occurrence of bleeding in both subgroups (Table 3).

**Table 3:** Summary of key efficacy and safety findings in randomized studies evaluating prolonged (>12 months) dual antiplatelet therapy (not specific to NSTEMI populations)

Trial [reference] ClinicalTrials.gov number	Patient population	Treatment and follow-up	Key efficacy findings	Key safety findings
TRA2°P-TIMI 50 [9] NCT00526474	History of atherothrombosis; an MI within previous 2–52 weeks	Vorapaxar (2.5 mg daily, *n* = 8898) versus placebo (*n* = 8881), both groups also received aspirin	Significant reduction in 3-year KM estimates for primary end point (CV death, MI, or stroke): 8.1 versus 9.7 %; HR, 0.80 (95 % CI, 0.72–0.89); *p* < 0.0001	Significant increase in 3-year KM estimates for moderate or severe bleeding: 3.4 versus 2.1 %; HR, 1.61 (95 % CI, 1.31–1.97); *p* < 0.0001
		Median follow-up = 2.5 years		
CHARISMA [13] NCT00050817	Prior MI, ischemic stroke, or PAD	Clopidogrel (75 mg daily, *n* = 4735) versus placebo (*n* = 4743), both groups also received aspirin	Significant reduction in primary end point (CV death, MI, or stroke): 7.3 versus 8.8 %; HR, 0.83 (95 % CI, 0.72–0.96); *p* = 0.01	No difference in rate of severe bleeding: 1.7 versus 1.5 %; HR, 1.12 (95 % CI, 0.81–1.53); *p* = 0.50 Significant increase in rate of moderate bleeding: 2.0 versus 1.3 %; HR, 1.60 (95 % CI, 1.16–2.20); *p* = 0.004
		Median follow-up = 27.6 months		
DAPT [46] NCT00977938	Had a coronary stent procedure (drug-eluting stent only)	After 12 months of clopidogrel or prasugrel plus aspirin, patients either continued on the thienopyridine (*n* = 5020) or received placebo (*n* = 4941) for another 18 months	Significant reduction in rates of: stent thrombosis (0.4 versus 1.4 %; HR, 0.29 [95 % CI, 0.17–0.48]; *p* < 0.001); major adverse CV and cerebrovascular events (4.3 versus 5.9 %; HR, 0.71 [95 % CI, 0.59–0.85]; *p* < 0.001); MI (2.1 versus 4.1 %; HR, 0.47 [95 CI: 0.37–0.61]; *p* < 0.001) Higher rate of all-cause mortality (2 versus 1.5 %; HR, 1.36 [95 % CI, 1.00–1.85]; *p* = 0.05)	Significant increase in rate of GUSTO moderate or severe bleeding: 2.5 versus 1.6 %, *p* = 0.001
DAPT subgroup analysis [47] NCT00977938	Had a coronary stent procedure (drug-eluting or bare-metal stents) following presentation with acute MI (*n* = 3576) or without evidence of MI (*n* = 8072)	After 12 months of clopidogrel or prasugrel plus aspirin, patients either continued on the thienopyridine (*n* = 5862) or received placebo (*n* = 5786) for another 18 months	Significant reduction in rates of: stent thrombosis (MI group: 0.5 versus 1.9 %; HR, 0.27 [95 % CI, 0.13–0.57]; *p* < 0.001; no MI group: 0.4 versus 1.1 %; HR, 0.33 [95 % CI, 0.18–0.60]; *p* < 0.001); major adverse CV and cerebrovascular events (MI group: 3.9 versus 6.8 %; HR, 0.56 [95 % CI, 0.42–0.76]; *p* < 0.001)	Significant increase in rate of GUSTO moderate or severe bleeding (MI group: 1.9 versus 0.8 %; HR, 2.38 [95 % CI, 1.28–4.43]; *p* = 0.005; no MI group: 2.6 versus 1.7 %; HR, 1.53 [95 % CI, 1.12–2.08]; *p* = 0.007)
			No significant reduction in rate of major adverse CV and cerebrovascular events in no MI group (4.4 versus 5.3 %; HR, 0.83 [95 % CI, 0.68–1.02); *p* = 0.08)	
ITALIC/ITALIC+ [49] NCT01476020	Had a coronary stent procedure (drug-eluting stent)	Patients randomized to either 6 months (*n* = 912) or 24 months (*n* = 910) of dual antiplatelet therapy post-stent, i.e. aspirin plus clopidogrel (75 mg/day) or prasugrel (60 mg/day) or ticagrelor (90 mg twice daily)	No significant difference in primary end point (death, MI, target lesion revascularization, stroke, and major bleeding at 12 months post-stent): 1.6 versus 1.5 %; HR, 1.072 (95 % CI, 0.517–2.221); *p* = 0.85 Non-inferiority demonstrated for 6- versus 12-month treatment; absolute risk difference 0.11 % (95 % CI, -1.04–1.26); *p* for non-inferiority = 0.0002	There were no significant differences in bleeding complications between the 6- and 24-month groups: Major bleeding occurred in only 3 (0.3 %) patients in the 24-month group (0 patients in 6-month group; HR, N/A); minor bleeding (0.5 versus 0.4 %; HR, 1.247 [95 % CI, 0.335–4.643]; *p* = 0.74
PEGASUS-TIMI 54 [51] NCT01225562	History of MI 1–3 years previously, at least one of the following risk factors: age ≥ 65 years, diabetes, a second prior MI, multivessel CAD that included ≥50 % occlusion in 2 or more coronary arteries, or chronic renal dysfunction	Three groups: ticagrelor (90 mg twice daily [*n* = 7050], or 60 mg twice daily [*n* = 7045]) and placebo (*n* = 7067); all groups also received aspirin	Significant reduction in 3-year KM estimates for primary end point (CV death, MI, or stroke): 7.85 % (90 mg ticagrelor; HR, 0.85 [95 % CI, 0.75–0.96]; *p* = 0.008), 7.77 % (60 mg ticagrelor; HR, 0.84 [95 % CI, 0.74–0.95]; *p* = 0.004), and 9.04 % (placebo)	Rates of TIMI major bleeding were higher with ticagrelor (90 mg: 2.60 %; 60 mg: 2.30 %) versus placebo (1.06 %; *p* < 0.001 for each ticagrelor dose)
	40.3 % of the overall patients had NSTEMI	Median follow-up: 33 months		

*CAD* coronary artery disease, *CI* confidence interval, *CV* cardiovascular, *GUSTO* Global Utilization of Streptokinase and TPA for Occluded Arteries, *HR* hazard ratio, *KM* Kaplan–Meier, *MI* myocardial infarction, *N/A* not applicable, *NSTEMI* non-ST-segment elevation myocardial infarction, *PAD* peripheral arterial disease, *TIMI* Thrombolysis in Myocardial Infarction

The potential for shortening the duration of dual anti-platelet therapy in patients following drug-eluting stent-PCI was evaluated in the ISAR-SAFE trial [48]. The trial results showed that the primary composite end point (death, MI, stent thrombosis, stroke or TIMI major bleeding) did not differ between the group treated for 6  versus 12 months (1.5 vs. 1.6 %, Δ −0.1 %, [1-sided 95 % CI, 0.5 %], *P*_noninferiority_ < 0.001). A trend toward lower rates of bleeding was observed in the group receiving 6 versus 12 months of dual antiplatelet therapy. Similar findings were observed in the ITALIC/ITALIC+ trial, which reported that for patients who respond well to aspirin, 6 months is non-inferior to 24 months of dual antiplatelet therapy for the composite primary end point of death, MI, target lesion revascularization, stroke, and major bleeding [49].

The multinational PEGASUS-TIMI 54 trial prospectively investigated the effect of aspirin and ticagrelor on outcomes in patients who had an MI 1–3 years previously [50, 51]. This trial included 21,162 high-risk patients, all currently taking low-dose aspirin (75–150 mg/day). As well as a history of MI, patients had at least one of the following risk factors: age ≥65 years, diabetes, a second prior MI, multivessel CAD that included ≥50 % occlusion in 2 or more coronary arteries, or chronic renal dysfunction. Patients were randomized 1:1:1 to placebo, ticagrelor 90 mg twice daily, or ticagrelor 60 mg twice daily. Median follow-up was 33 months. Both ticagrelor doses significantly reduced the primary efficacy end point (composite of cardiovascular death, MI, or stroke), compared with placebo. At 3 years, the Kaplan–Meier rates were 7.85 % (ticagrelor 90 mg), 7.77 % (ticagrelor 60 mg), and 9.04 % (placebo); ticagrelor 90 mg: HR, 0.85 (95 % CI, 0.75–0.96]; *p* = 0.008; ticagrelor 60 mg: HR, 0.84 (95 % CI, 0.74–0.95]; *p* = 0.004. Rates of TIMI major bleeding (primary safety end point) were higher with ticagrelor (90 mg: 2.60 %; 60 mg: 2.30 %) versus placebo (1.06 %; *p* < 0.001 for each ticagrelor dose) [50]. The PEGASUS-TIMI 54 data demonstrate the potential benefit of dual antiplatelet therapy (ticagrelor and aspirin) beyond 12 months in high-risk, post-MI patients. Although this trial was not specific for patients with NSTEMI, at baseline, 40.3 % of the overall patients had NSTEMI.

Of the five randomized studies evaluating prolonged (>12 months) dual antiplatelet therapy, four studies (including one subgroup analysis) demonstrated significant clinical benefit (reduction in the primary efficacy end point) with extending treatment, compared with controls. Although in four studies, prolonged treatment resulted in increased bleeding (Table 3). None of these trials were specific for patients with NSTEMI, thus highlighting the need for further research. Subanalyses of NSTEMI patients in PEGASUS-TIMI 54, and more prospective studies in high-risk NSTEMI patients, will advance our understanding of long-term management of these patients.

## Conclusions

There are currently limited data to guide clinical decision making around optimal secondary preventive therapies in NSTEMI patients who survive 12 months or more after MI. While 12 months appears to be the optimal duration of dual antiplatelet therapy for most patients, this may not be the case for high-risk patients. Ongoing risk assessment (for bleeding and ischemia) is important in all post-MI patients, and clinicians should use objective measures of assessment whenever possible to avoid over- or under-estimating future risk. Physicians also need to regularly assess the risks and benefits of all therapies to suit the patient’s clinical status, which may change over time in the years following ACS. More research is urgently needed to help guide therapeutic decision making during long-term management of complex patients after NSTEMI.
